# Medical Society of the County of New York

**Published:** 1885-01

**Authors:** 


### Medical Society of the County of New York.

Dr. Daniel Lewis, one of the most prominent physicians of the metropolis, has been elected President of this society for the ensuing year. At an adjourned annual and stated meeting, held Nov. 24, 1884, he delivered an address, in the course of which he remarked that the law of 1880 regulating the practice of medicine in the State of New York had proved more far-reaching and effective than even its framers had anticipated. In regard to a State Medical Examining Board, he said that its necessity as a means of suppressing quackery and promoting a higher grade of medical education was obvious. Such a Board, however, should it be organized, as was probable in the near future, he thought should have power to revoke a license to practice for sufficient reasons. These remarks possess an additional significance, from the fact that Dr. Lewis is a member of the standing committee on legislation of the State Medical Society. This committee, together with Drs. A. L. Loomis, J. G. Curtis, and A. Van Derveer, were, at the last annual meeting, constituted a special committee to draft a bill on the subject of a Board of Medical Examiners, and to report the same at the next annual meeting, which will occur February 3, 1885. By the terms of the resolution creating this committee, it was directed to communicate with all the medical societies throughout the State on the subject.

The composition of this committee is such as to justify the expectation that a bill will be reported satisfactory to all the interests involved; and we anticipate, as the result of its labors, a good beginning in the direction of a higher standard for the degree of the doctorate in medicine in this State.

